# Computed tomographic coronary angiography for patients with heart failure (CTA-HF): a randomized controlled trial (IMAGE HF Project 1-C)

**DOI:** 10.1186/1745-6215-14-443

**Published:** 2013-12-26

**Authors:** Benjamin JW Chow, Rachel E Green, Doug Coyle, Mika Laine, Helena Hanninen, Hanna Leskinen, Miroslav Rajda, Eric Larose, Juha Hartikainen, Marja Hedman, Lisa Mielniczuk, Eileen O’Meara, Robert A deKemp, Ran Klein, Ian Paterson, James A White, Seppo Yla-Herttuala, Alex Leber, Vikas Tandon, Ting Lee, Abdul Al-Hesayen, Renee Hessian, Taylor Dowsley, Malek Kass, Cathy Kelly, Linda Garrard, Jean-Claude Tardif, Juhani Knuuti, Rob S Beanlands, George A Wells

**Affiliations:** 1 Department of Medicine, Division of Cardiology, (including Cardiac Imaging, The Heart Failure Program, and the Cardiac ResearchMethods Centre), University of Ottawa Heart Institute, 40 Ruskin Street, Ottawa, ON K1Y 4W7, Canada; 2 Department of Medicine, University of Ottawa, 75 Laurier Avenue East, Ottawa, ON K1N 6N5, Canada; 3 Department of Radiology, University of Ottawa, 75 Laurier Avenue East, Ottawa, ON K1N 6N5, Canada; 4 Department of Epidemiology and Community Medicine, University of Ottawa, 75 Laurier Avenue East, Ottawa, ON K1N 6N5, Canada; 5 Helsinki University Central Hospital, Stenbäckinkatu 9, PO BOX 100, FI-00029, Helsinki, Finland; 6 Turku PET Centre, Turku PET Centre, c/o Turku University Hospital, P.O. Box 5220521, Turku, Finland; 7 Dalhousie University, 6299 South Street, PO Box 15000, Halifax NS B3H 4R2, Canada; 8 Université de Québec, 2325 Rue de l'Université, Québec City, QC G1V 0B4, Canada; 9 Heart Centre Kuopio University Hospital, 2 Puijonlaaksontie, 70211, Kuopio, Finland; 10 Montréal Heart Institute, Université de Montréal, 5000 Bélanger Street, Montréal, QC H1T 1C8, Canada; 11 University of Alberta, 116 St and 85 Ave, Edmonton, AB T6G 2R3, Canada; 12 London Health Sciences Centre, 339 Windermere Road, London, ON N6A 5A5, Canada; 13 Sunnybrook Health Sciences Centre, 2075 Bayview Ave, Toronto, ON M4N 3M5, Canada; 14 McMaster University, 1280 Main St W, Hamilton, ON L8S 4L8, Canada; 15 University of Manitoba, 66 Chancellors Cir, Winnipeg, MB R3T 2N2, Canada

## Abstract

**Background:**

The prevalence of heart failure (HF) is rising in industrialized and developing countries. Though invasive coronary angiography (ICA) remains the gold standard for anatomical assessment of coronary artery disease in HF patients, alternatives are being sought. Computed tomographic coronary angiography (CTA) has emerged as an accurate non-invasive diagnostic tool for coronary artery disease (CAD) and has been demonstrated to have prognostic value. Whether or not CTA can be used in HF patients is unknown. Acknowledging the aging population, the growing prevalence of HF and the increasing financial burden of healthcare, we need to identify non-invasive diagnostic tests that are available, safe, accurate and cost-effective.

**Methods/Design:**

The proposed study aims to provide insight into the efficacy of CTA in HF patients. A multicenter randomized controlled trial will enroll 250 HF patients requiring coronary anatomical definition. Enrolled patients will be randomized to either CTA or ICA (n = 125 per group) as the first test to define coronary anatomy. The primary outcomes will be collected to determine downstream resource utilization. Secondary outcomes will include the composite clinical events and major adverse cardiac events. In addition, the accuracy of CTA for detecting coronary anatomy and obstruction will be assessed in patients who subsequently undergo both CTA and ICA. It is expected that CTA will be a more cost-effective strategy for diagnosis: yielding similar outcomes with fewer procedural risks and improved resource utilization.

**Trial registration:**

ClinicalTrials.gov, NCT01283659

Team grant #CIF 99470

## Background

The prevalence of heart failure (HF) is on the rise in both industrialized and developing countries [1-3]. Though the diagnosis of HF often confers a poor prognosis, patient outcomes vary widely according to the underlying etiology of HF and subsequent therapy [2]. It has been long accepted that patients with ischemic cardiomyopathy and significant viable myocardium may derive mortality benefit from revascularization [2]. As such, invasive coronary angiography (ICA) is often recommended in patients with HF to define the presence or absence of coronary artery disease (CAD) as the inciting etiology.

ICA remains the gold standard for the anatomical assessment of coronary arteries and the diagnosis of luminal stenoses. However, access to ICA in Canada and other countries is limited, costly and has inherent risks (death (0.12%), myocardial infarction (<0.05%), stroke (0.1%) and bleeding (1.6%)), which prohibit its routine use in all patients. A non-invasive alternative to ICA would be desirable if it were available, accurate, safe, and cost-effective.

Computed tomographic coronary angiography (CTA) has emerged as an accurate non-invasive diagnostic tool for CAD [4-8] and has been demonstrated to have prognostic value [9-11]. Whether or not CTA can be used cost-effectively in HF patients for diagnosis and to guide patient investigations and management is unknown. This is the focus of this randomized controlled trial (RCT).

To date, there are limited randomized controlled trials (RCTs) that study the role of non-invasive imaging for the diagnosis and management of patients with HF [12]. To our knowledge, there are no RCTs examining the utility of CTA in HF patients. Acknowledging the aging population, the growing prevalence of HF and the increasing financial burden of healthcare [1-3], we need to identify non-invasive diagnostic tests that are cost-effective, readily available and safe, and of sufficient accuracy to risk stratify patients and guide investigations and management.

**Figure 1:**
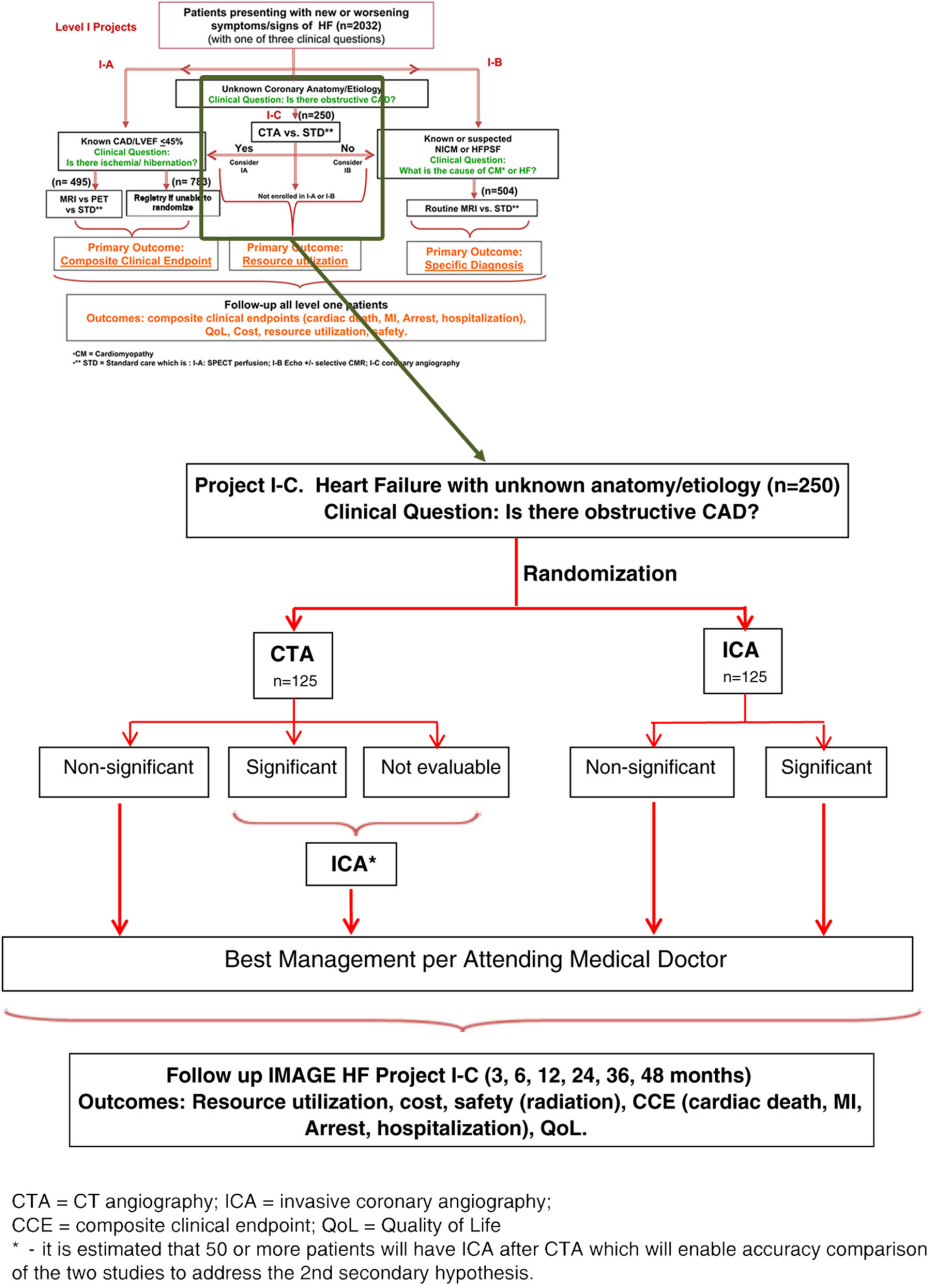
**IMAGE-HF Project I-C: experimental algorithm to evaluate use of tomographic coronary angiography (CTA) in determination of heart failure (HF) etiology.** Figure 1 illustrates the experimental model of this study, in which the ability of CTA to determine etiology of HF will be compared to that of invasive coronary angiography (ICA). All patients exhibiting HF of unknown etiology will be considered; patients with contraindications to CTA will be excluded.

In 2007, the Canadian Institutes of Health Research New Frontiers Program consensus conference identified the need for comparative effectiveness research evaluation of imaging, particularly in HF. In response, the IMAGE-HF team project has been established The main goal of IMAGE-HF is to evaluate cardiac imaging in HF. Studies are divided into three broad levels: Level 1) research that compares the effective use of existing clinical practice strategies for HF to determine impact on relevant outcomes; Level 2) research to evaluate emerging methods to address specific HF populations; Level 3) research for the development and evaluation of novel imaging biomarkers in animal models of HF for potential translation to human studies. Level 1 projects comprise three independent trials: Project 1-A and 1-B are described separately [13,14]. The study herein is an IMAGE-HF Project 1-C called: *Computed Tomographic Coronary Angiography for Patients with Heart Failure (CTA-HF): A Randomized Controlled Trial.* This study will compare the resource utilization (downstream ICA, other testing and/or cardiac hospitalization) in terms of average health care costs per patient of CTA compared to ICA in patients with progressive or newly diagnosed HF (Figure 1).

### Objectives

The primary objective of this RCT is to compare the average health care costs of CTA (experimental algorithm) to ICA (standard of care) (Figure 1) as the initial test for coronary anatomy in HF patients of unknown etiology. The secondary objectives are to compare CTA versus ICA for composite clinical events, major adverse cardiac events (MACE), quality of life (QoL), radiation exposure and safety.

### Hypotheses

The primary hypothesis is that, compared to ICA, a diagnostic strategy algorithm using CTA for HF patients will result in a reduction in downstream resource utilization leading to a reduction in average costs per patient.

The first of two secondary hypotheses states that compared to ICA (standard of care), a CTA strategy will achieve: a) similar composite clinical events (CCE) (cardiac death, myocardial infarction (MI), resuscitated cardiac arrest and cardiac re-hospitalization (worsening heart failure, acute coronary syndrome arrhythmia), QoL, and MACE (cardiac death, non-fatal MI); b) a lower rate of procedure related complications (death, MI, stroke, vascular complications, severe allergic reactions; contrast nephropathy); and c) a lower rate of normal ICA. The second of the two secondary hypotheses states that using patient-based analysis and vessel-based analysis, CTA has very good agreement with ICA among patients with HF in the CTA arm who proceed to ICA.

## Methods/Design

### Design

CTA-HF is a multicenter, multinational, randomized controlled trial that is currently recruiting 250 patients from 15 sites across Canada and Finland (see Additional file 1) over 4 years, randomized to CTA versus ICA (n = 125 per group) as the first test to define coronary anatomy in patients with heart failure. This research study has approval from each participating center’s institutional research ethics board.

### Eligibility criteria

All HF patients requiring investigation to determine the etiology of HF (ischemic versus non-ischemic) will be screened for the study. Eligible patients will have a documented history of left ventricular dysfunction (LVEF <50% by radionuclide angiography (RNA), echocardiography or magnetic resonance imaging (MRI)), or New York Heart Association (NYHA) Class II to IV symptoms, or an admission to hospital or emergency room for heart failure within the past 12 months. As well, the HF patient requires diagnostic information on coronary anatomy because the diagnosis of CAD is uncertain or the definition of coronary anatomy is required for management.

Patients will be excluded if they have: age <18 years, lack of consent, renal insufficiency (glomerular filtration rate, GFR <45 ml/min), allergy to intravenous contrast agents, contraindications to radiation exposure (for example, pregnancy), uncontrolled heart rate (HR, as per local clinical routine), history of revascularization, atrial fibrillation, frequent atrial or ventricular ectopy (>1/minute), inability to perform 20-second breath-hold, and CTA or ICA within the preceding 12 months.

### Randomization

Randomization will be coordinated by the University of Ottawa Heart Institute (UOHI) Cardiovascular Research Methods Centre. Eligible, consenting patients will be randomized to either the CTA or ICA arm and stratified according to recruitment site. A stratified block (varying sizes) randomization scheme will be used. Within each stratum, patients will be randomized with varying block sizes into the two study groups. A central randomization scheme, ensuring concealment, will be used and the site coordinator will perform patient assignments. The randomization scheme will be generated by the study statistician using a SAS (SAS Institute, Cary, North Carolina, USA) macro.

### Data collection

All data will be collected using the Autonomy Process Automation (AMA) formally called Liquid Office™, which is a web-based solution for creating, routing, data capturing and managing electronic forms. This will be done in conjunction with the UOHI Cardiovascular Research and Methods Centre and will be audited using standard procedures.

Recruiting centers will be responsible for patient screening and local enrollment. Identification of patients will be coordinated by physicians and support staff responsible for scheduling coronary angiography. Local research staff (under direction from the site principal investigator) will identify and recruit patients for the study as well as be responsible for the day-to-day management of the study. A supervising research coordinator, based at the UOHI, will oversee the multicenter study at all sites and routine site visits will be made.

### Interventions

Patients will be randomized to CTA (experimental algorithm) versus ICA (standard of care).

#### Computed tomographic coronary angiography

Non-contrast enhanced prospective ECG-triggered image acquisition will be acquired and the Agatston score will be calculated [15]. Standard procedures will be applied for contrast infusion, timing and image acquisition. As per local clinical practice, prospective ECG-triggered or retrospective ECG-gated data sets will be acquired using bi- or tri-phasic contrast protocols. Vendor-specific radiation dose-reducing techniques such as prospective ECG-triggered image acquisition will be encouraged. A 4-point grading score [normal, mild (<50%), moderate (50 to 69%), severe (≥70%)] will be used for the evaluation of coronary stenosis using a 17-segment model. Similar to ICA, obstructive CAD is defined as coronary diameter stenosis ≥50% (Additional file 2).

#### Invasive coronary angiography

ICA will be performed according to standard clinical protocols, with selective coronary injection and images acquired from multiple oblique views (Additional file 2).

Patients with obstructive CAD will be categorized as having high risk CAD (defined as having a left main stenosis (≥50%), or three-vessel disease (VD) (≥70%) or two-VD (≥70%) involving the proximal left anterior descending artery) or non-high risk CAD [9].

Following either CTA or ICA, information on the results will be disseminated to the referring physician as part of their standard clinical care.

### Outcome measures

The primary outcome (average health care costs per patient) will be determined through regression methods detailed below.

All secondary outcome measures will be determined: (CCE (cardiac death, myocardial infarction, resuscitated cardiac arrest and cardiac re-hospitalization (worsening heart failure, acute coronary syndrome arrhythmia), QoL, procedural complications (all-cause death, myocardial infarction, stroke, vascular complications, severe allergic reactions; contrast nephropathy), and rate of normal ICA.

CTA accuracy: To address the 2^nd^ secondary hypothesis, regarding CTA accuracy, the accuracy in the cohort of patients randomized to CTA and undergoing subsequent ICA (sensitivity, specificity, predictive values and likelihood ratios) will be determined and reported with 95% confidence intervals (CI). It is estimated that 50/125 of the patients randomized to CTA will have subsequent ICA, which will enable the comparison of CTA accuracy to ICA.

### Frequency and duration of follow-up

**Figure 2:**
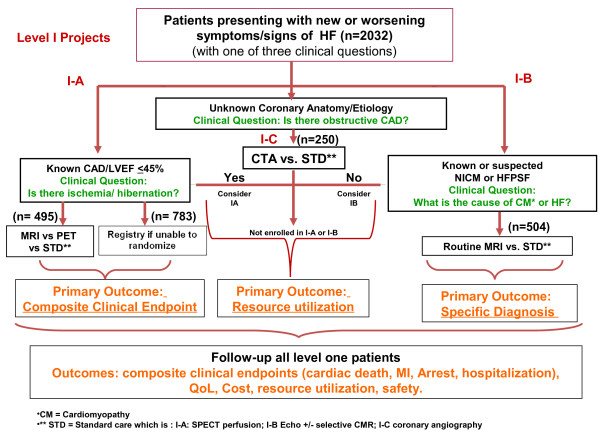
Timeline for IMAGE-HF Project I-C patient follow-up.

Telephone follow-up will be performed at 3, 6 and 12 months, and every 12 months thereafter. Follow-up will continue until the termination of the trial (mean = 3 years; range 2 to 4 years) (Figure 2).

### Safety and ethics

The study protocol will be approved by each institution’s research ethics board and informed consent form will be obtained from enrolled patients. This study will be conducted according to the Declaration of Helsinki, Good Clinical Practice and the TriCouncil Policy.

### Sample size

Typically, sample sizes for economic analyses are based upon practical and logistic reasons with limited focus on inferential statistics [16]. Within this study, incremental costs will be estimated through regression analysis, which will incorporate a significant number of covariates to adjust for the nonrandom nature of treatment allocation. Based on traditional sample size calculations for regression analysis, a sample size of 250 will be sufficient to control for a large number of coefficients (at least 25) and give a suitable degree of precision around our primary outcome [17]. For example, if the coefficient of variation for costs was 25%, there would be sufficient power to detect a reduction in costs by 10%.

### Data analysis

For the purposes of data analysis, two study groups will be defined: ‘intention-to-treat’ (ITT) population which consists of all patients who are randomized to the CTA or ICA regardless of whether they actually receive the approach, and ‘per protocol’ population which excludes patients randomized but not receiving the allocated approach. Primary analyses will be conducted on the ITT population, and the sensitivity analyses will be conducted on the per-protocol population. Descriptive statistics for the baseline demographic characteristics will be summarized for the study group by means of frequency for categorical variables and mean, standard deviation, median and interquartile ranges for continuous variables. Demographic information includes age, comorbidities, cardiac history, cardiac risk factors and medications. Groups will be compared on continuous variables using Wilcoxon rank-sum tests and on categorical variables using Fisher’s exact tests.

### Primary analysis

Primary analysis will estimate average health care costs per patient for CTA versus ICA. Resource utilization will be measured as detailed below. Cost is defined as the incremental cost of the diagnostic strategy using CTA and will represent the primary outcome measure. Cost will be estimated through regression methods detailed below. Thus, estimations of incremental cost will be evaluated based on costing the components of care.

### Secondary analysis

Secondary analysis will investigate CCE and MACE, analyzed using survival analysis techniques between the ICA and CTA arms. The survival experience (that is, time-to-major adverse cardiac event) in each of the two arms will be summarized using Kaplan-Meier product limit estimates and the log-rank test will be used for comparing the survival curves. The hazard ratio with 95% confidence interval (CI) will be calculated. To adjust for possible effects of covariates on survival, the Cox’s proportional hazards model will be used. Underlying assumptions for these statistical procedures will be assessed; in particular, the proportional hazard assumption will be assessed using graphical and numerical tests, and stratified Cox models or Aalen’s additive model will be considered as needed.

#### Safety

The proportion of various procedural complications will be compared descriptively using chi-square tests to determine relative safety of CTA to ICA. Patient morbidity/health-related QOL associated with CTA and/or ICA will be compared using Student-t tests for the overall scores and utility.

The accuracy (operating characteristics) of CTA in the identification of high-risk coronary anatomy and obstructive CAD (per-patient/vessel/segment analysis) will be assessed in the cohort of patients undergoing both CTA and ICA. A receiver operating characteristic (ROC) curve analysis will be conducted for the vessel disease number, and the weighted kappa with 95% CI will be calculated to assess the agreement between CTA and ICA for the allocation of patients into the groups: 0-, 1-, 2- or 3-vessel disease; high-risk CAD; and non-high risk CAD. ROC analysis will also be performed on a per-patient, per-vessel and per-segment basis. In the patients who undergo both CTA and ICA, the accuracy of CTA to predict subsequent early revascularization will be assessed as follows: the point estimates with 95% CI for the sensitivity (S_e_), specificity (S_p_), positive predictive value (PPV), and negative predictive value (NPV) of CTA in identifying a patient for revascularization (medical therapy (no obstructive CAD) versus revascularization (PCI (non-high risk CAD) versus CABG (high-risk CAD)) using ICA as the gold standard will be calculated. They will be adjusted for referral bias using the Diamond method [18].

### Cost analysis

Analysis will take the form of a simple cost minimization analysis comparing a management algorithm using CTA versus ICA. Analysis will be restricted to the follow-up period within the study database and will be conducted from the health care system perspective.

### Costs

Costs can be grouped into four main categories: initial imaging modalities, medications, hospitalizations and ongoing patient management. The total costs for each patient will be estimated through a simple two-stage process; measurement and valuation of resource use. Following this, the incremental costs associated with the use of CTA will be derived through regression modelling.

#### Measurement of physical quantities of resources consumed

As part of the database constructed for this project, data on the use of health care resources are recorded. Specific resources for which information will be collected are initial imaging modalities, specialist outpatient appointments, family physician consultations, medications, further testing and investigations including invasive coronary angiography and hospitalizations.

#### Valuation of resource use

For all resource items listed above an appropriate unit cost will be derived. Unit costs will be obtained from appropriate provincial fee schedules and administrative data as for project 1A.

#### Costs associated with the use of computed tomographic coronary angiography

The incremental cost associated with the management algorithm that includes the use of CTA will be estimated as follows. A data set will be constructed containing the total costs per patient. For each observation the patient will be assigned a dummy variable relating to whether they were assigned to the management algorithm or to standard care. Regression will be conducted using appropriate generalized linear models controlling for all potential covariates. The appropriate coefficient from the regression will represent the incremental cost associated with the use of CTA.

### Analysis of uncertainty

Given the complex nature of cost data, generalized linear models (GLMs) will be adopted to determine the incremental costs associated with CTA. GLMs are attractive in the analysis of cost data as these models involve a parametric method where non-normal distributions can be specified and the way the dependent variable and the independent variables interact can be altered [18]. GLMs involve a link function that specifies the relationship between the mean and the covariates and a family, which specifies the assumed mean-variance relationship. Different families and link functions are available. The modified Parks test can be used to determine the appropriate family while the novel technique of extended estimating equations has been developed, which allows identification of both the link function and family [18-20]. The regression model will provide 95% confidence intervals around the estimate of incremental costs. Univariate sensitivity analysis will be conducted to assess the robustness of the study’s results to changing assumptions related to the unit costs of specific resource items. In addition, where available, sensitivity analysis will be conducted utilizing resource use data.

### Missing data

‘Missingness’ is considered to be missing at random (MAR) and mixed methods repeated measures (MMRM) and multiple imputation (MI) techniques will be used for handling missing data. In particular, for continuous outcomes at multiple time points, MMRM will be used.

### Study management

The IMAGE-HF trial is managed by an Executive Committee consisting of clinicians specialized in diagnostic imaging and heart failure and experts in biostatistics, physics and radiochemistry, as well as a larger Steering Committee consisting of members of the Executive Committee and representatives of all the initial study centers. In addition, an events adjudication committee (blinded to patient allocation) will independently review and adjudicate each clinical event. Since all the imaging approaches are part of standard clinical practice, no interim analysis is planned, but there will be independent data safety monitoring board (DSMB), which will review the safety data on a periodic basis.

## Discussion

The proposed RCT is the largest RCT in HF patients to compare CTA versus ICA. This RCT will accomplish several goals: 1) it will determine feasibility and diagnostic accuracy of CTA in patients with undifferentiated heart failure; 2) if feasible, it will determine if the incorporation of CTA into the investigation algorithm of HF patients is cost-effective; and 3) it will determine if the CTA strategy, compared to ICA, is safe.

If these hypotheses are supported, study findings will have significant implications related to clinical practice. Evidence that CTA is effective in distinguishing etiology of HF at a lower cost with reductions in unnecessary ICA testing, subsequently with fewer procedure-associated complications, would be very attractive to clinicians and patients. This may further contribute to a reduction in healthcare costs and reduce current burdens on healthcare systems worldwide.

## Trial status

This randomized clinical trial is currently recruiting subjects.

## Abbreviations

CAD: Coronary artery disease; CCE: Composite clinical events; CTA: Tomographic coronary angiography; DSMB: Data safety monitoring board; GFR: Glomerular filtration rate GLMs, generalized linear models; HF: Heart failure; HR: heart rate; ICA: Invasive coronary angiography; ITT: Intention-to-treat; MACE: Major adverse cardiac events; MRI: Magnetic resonance imaging; QoL: Quality of life; RCT: Randomized controlled trial; RNA: Radionuclide angiography; ROC: Receiver operating characteristic; VD: Vessel disease.

## Competing interests

B Chow receives research and fellowship training support from GE Healthcare, research support from Pfizer and AstraZeneca, and educational support from TeraRecon Inc. E O’Meara receives research funding from Johnson & Johnson for the Cardiorenal-anemia syndrome in HF. R deKemp is a consultant for Jubilant DraxImage and receives research funding from Lantheus Medical Imaging, GE, MDS Nordion and receives revenues from rubidium generator technology licensed to Jubilant DraxImage and receives revenues from FlowQuant software sales. Ran Klein is a consultant Jubilant DraxImage and receives revenues from rubidium generator technology licensed to Jubilant DraxImage and receives revenues from FlowQuant software sales. Alex Leber receives research support from Toshiba for CT work. R Beanlands is a consultant for Lantheus Medical Imaging, DraxImage and receives research funding from Lantheus Medical Imaging, GE, MDS Nordion. All other authors declare that they have no competing interests.

## Authors’ contributions

BC, RB, GW, JK conceived of this study, and participated in its design, coordination and helped to draft the manuscript. LG is involved in the study design and project management and helped to draft the manuscript. RdK and RK established and will monitor the standardization of the imaging modalities; DC designed and will coordinate the economic evaluation. The following contributed to design of trial and will be involved with conducting the trial: RG, ML, HH, HL, MR, EL, JH, MH, LM, EO, IP, JAW, SY-H, AL, VT, TL, AA-H, RH, TD, MK, CK, JCT). All authors read and approved the final manuscript.

## Supplementary Material

Additional file 1: Table S1 List of IMAGE-HF participating sites and investigators. Click here for file

Additional file 2 Standardization and quality assurance. Click here for file
