# Supplementary material for: Computed tomographic coronary angiography for patients with heart failure (CTA-HF): a randomized controlled trial (IMAGE HF Project 1-C)
Source: Trials. 2013 Dec 26;14:443. doi: 10.1186/1745-6215-14-443 (PMC3895694; doi:10.1186/1745-6215-14-443)
Supplement: Additional file 1: Table S1 — List of IMAGE-HF participating sites and investigators. [file 1745-6215-14-443-S1.doc]

**Additional file 1: Table S1. List of** IMAGE-HF participating sites and investigators

|  |  |
| --- | --- |
| University of Ottawa Heart Institute |  |
| R Beanlands | Co-Principal Investigator IMAGE-HF, Canada ^a^ |
| G.A. Wells | Principal Investigator CRMC ^a^ |
| R. deKemp | Principal Investigator QA Program ^a^ |
| D. Birnie | Co-Principal Investigator Project IIA |
| L. Mielniczuk | Co-Principal Investigator Project IA |
| K. Chan | Site Principal Investigator |
| B. Chow | Principal Investigator Project IC |
| L. Garrard | Project Management |
| R. Hessian | Investigator |
| T. Ruddy | Investigator |
| R.A. Davies | Investigator |
| H. Haddad | Investigator |
| A. Dick | Investigator |
| C. Dennie | Investigator |
| D. Coyle | Investigator |
| B. McArdle | Investigator |
| T. Dowsley | Investigator |
| G.Dwivedi | Investigator |
| A. Al-Hesayen | Investigator |
| J. DaSilva | Investigator |
| C. Kelly | Research Coordinator |
| E. Moga | Research Coordinator |
| R. Klein | Core Lab Manager |
| K. Williams | Statistician |
| R. Fleming | Research Coordinator |
| M. Boomgaardt | Research Coordinator |
| Montreal Heart Institute-Université de Montréal |  |
| J.C. Tardif | Investigator a |
| E. O’Meara | Co-Principal Investigator Project IA |
| M. Friedrich | Investigatora |
| J. Rouleau | Investigator |
| T. Heinonen | Investigator |
| F. Marcotte | Investigator |
| N. Racine | Investigator |
| H.Q. Ly | Investigator |
| J. Morrissette | Research Coordinator |
| H. Brown | Research Coordinator |
| University of Alberta |  |
| I. Paterson | Principal Investigator Project IB |
| L. Lalonde | Investigator |
| J. Ezekowitz | Investigator |
| M. Irwin | Research Coordinator |
| University of Turku |  |
| J. Knuuti | Co-Principal Investigator IMAGE-HF, Finland a |
| H. Ukkonen | Investigator |
| S. Yla-Herttuala | Investigator a |
| H. Leskinen | Investigator |
| A. Saraste | Investigator |
| T. Vasankari | Research Coordinator |
| K. Lahtonen | Research Coordinator |
| M. Tarkia | Site Project Manager |
| University Central Hospital, Helsinki |  |
| M. Laine | Site Principal Investigator |
| H. Hanninen | Investigator |
| M. Pietila | Research Coordinator |
| Heart Centre, Kuopio University Hospital |  |
| J. Hartikainen | Site Principal Investigator |
| M. Hedman | Investigator |
| S. Karkkainen | Investigator |
| I. Kaivonurmi | Research Coordinator |
| M. Sutinen | Research Coordinator |
| Sunnybrook Health Sciences Centre |  |
| G. Wright | Site Co-Principal Investigator a |
| K. Connelly | Site Co-Principal Investigator |
| R. Myers | Investigator |
| C. Cunningham | Investigator |
| E. Crystal | Investigator |
| A. Leber | Investigator |
| M. Mohammed | Research Coordinator |
| J. Malko | Research Coordinator |
| University of Calgary |  |
| A. Howarth | Site Co-Principal Investigator |
| T. Anderson | Site Co-Principal Investigator |
| A. Krysk | Investigator |
| S. Hutchison | Investigator |
| N. Merchant | Investigator |
| S. Weeks | Investigator |
| R. Sandonato | Research Coordinator |
| S. Rivest | Research Coordinator |
| J. Veenhuyzen | Research Coordinator |
| M. Seib | Research Coordinator |
| B. Madden | Research Coordinator |
| D. Durand | Research Coordinator |
| London Health Sciences |  |
| M. Arnold | Site Principal Investigator a |
| G. Wisenberg | Investigator |
| T. Lee | Investigator |
| F. Prato | Investigator |
| J. White | Co-Principal Investigator Project IIA |
| K. Carter | Research Coordinator |
| Laval University |  |
| E. Larose | Site Principal Investigator |
| P. Pibarot | Investigator a |
| B. Cantin | Investigator |
| J. Carange | Research Coordinator |
| K. Bibeau | Research Coordinator |
| St. Michael’s Hospital |  |
| M. Freeman | Site Co-Principal Investigator |
| K. Connelly | Site Co-Principal Investigator |
| H. Leong-Poi | Investigator |
| G. Moe | Investigator |
| A. Al-Hesayen | Investigator |
| J. Sloninko | Research Coordinator |
| Hamilton |  |
| V. Tandon | Site Principal Investigator |
| K. Gulenchyn | Investigator |
| F. Spence | Investigator |
| A. Khoorshed | Research Coordinator |
| Sherbrooke |  |
| E. Turcotte | Site Principal Investigator |
| S. Lepage | Investigator |
| Paul Farand | Investigator |
| S. Joncas | Resident, recruitment |
| E. Lavallee | Research Coordinator |
| Halifax |  |
| M. Rajda | Site Principal Investigator |
| R. Stewart | Investigator |
| J. Clarke | Investigator |
| S. Burrell | Investigator |
| B. Clarke | Investigator |
| S. Yarn | Research Coordinator |
| M. MacFarlane | Research Coordinator |
| Winnipeg |  |
| M. Kass | Site Principal Investigator |
| J. Tam | Investigator |
| T. Moore | Research Coordinator |
| A. Munoz | Research Coordinator |
| QA Core Labs |  |
| R. deKemp | Investigator - QA and Standardization Lead |
|  | PET, SPECT QA Core Lab Team Leader (Ottawa) |
| R. Klein | Core Lab Manager (Ottawa) |
| B. McArdle | PET, SPECT QA Core Lab (Ottawa) |
| J. Renaud | PET, SPECT QA Core Lab (Ottawa) |
| K. Chan | ECHO QA Core Lab Team Leader (Ottawa) |
| J. White | CMR QA Core Lab 1A Team Leader (London) |
| I. Pauchard | CMR QA Core Lab 1A (London) |
| I. Patterson | CMR QA Core Lab 1B Team Leader (Edmonton) |
| P. L’Allier | ICA QA Core Lab Team Leader (Montreal) |
| B. Chow | CTA QA Core Lab Team Leader (Ottawa) |
|  |  |
|  |  |
|  |  |
|  |  |
|  |  |
|  |  |
|  |  |
|  |  |
|  |  |
|  |  |
|  |  |
| DSMB |  |
| A. Krahn, Chair |  |
| J. Fallavollita |  |
| L. Thabane |  |
| Events |  |
| H. Haddad, Chair |  |
| D.S. Beanlands |  |
| L. Duchesne |  |
| J. Ezekowitz |  |
| R.A. Davies |  |

^a^ Steering Committee members.
