# Supplementary material for: Computed tomographic coronary angiography for patients with heart failure (CTA-HF): a randomized controlled trial (IMAGE HF Project 1-C)
Source: Trials. 2013 Dec 26;14:443. doi: 10.1186/1745-6215-14-443 (PMC3895694; doi:10.1186/1745-6215-14-443)
Supplement: Additional file 2 — Standardization and quality assurance. [file 1745-6215-14-443-S2.doc]

Additional file 2: Standardization and quality assurance (IMAGE-QA)

The IMAGE-HF QA program standardizes several important aspects of the clinical imaging research program:

1. defining best current imaging practice for standard-care tests

2. disseminating advanced imaging technology and standards

3. promoting structured reporting and comprehensive imaging QA

4. ensuring consistent interpretation and patient management recommendations

For the IMAGE-HF project 1C (CTA-HF), this includes standard operating procedures (SOPs) for cardiac CT angiography (CTA) and invasive coronary angiography (ICA), as well as structured reporting elements and quality assurance review by QA-CORE labs (SOPs and CRFs are posted on the IMAGE-HF website).

1. Standard-care Imaging Protocols

Invasive Coronary Angiography (ICA):

IMAGE-HF-1C SOP CATH-anatomy

Protocols will include selection and recording of catheters, projection views, lesion assessment based on revised NIH nomenclature, QCA image analysis and reporting. {AU Query: Please cite publications using numbers that indicated placement of reference in reference section rather than using author names as above.}

2. Advanced Imaging Protocols

Coronary CT Angiography (CTA):

IMAGE-HF-1C SOP CT-anatomy

Cardiac CT protocols will include instructs regarding heart-rate control, contrast administration, angiography and calcium imaging, as well as CTA image analysis, lesion assessment and reporting.

3. Common Structured Reporting Elements

Invasive Coronary Angiography (ICA):

The following parameters are included on the ICA interpretation CRF: stenosis severity in 17 standard coronary artery segments, wall-motion and LV systolic function parameters if available. Clinical interpretation of the number of diseased vessels, high-risk classification, recommended clinical management for revascularization or medical therapy, and confirmation that the recommendation was communicated to the referring physician are captured on the CRF.

Coronary CT Angiography (CTA):

The following common parameters are included on the CTA interpretation CRF: stenosis and plaque severity in 17 standard coronary artery segments, wall-motion and LV systolic function parameters if available. Clinical interpretation of the number of diseased vessels, high-risk classification, recommended clinical management for angiography or medical therapy, and confirmation that the recommendation was communicated to the referring physician are captured on the CRF.

4. Quality Assurance CORE lab reviews (QA-CORE)

A subset of scans (10%) is targeted for co-reading interpretation at an experienced site identified as the CORE lab for each imaging modality. The first 2 scans (and 5% of the subsequent scans) from each imaging modality at each recruiting site are transferred to the corresponding modality QA-CORE lab for clinical interpretation and comparison to the site interpretation for quality assurance. Disagreements in the overall interpretation of the number of diseased vessels, high-risk classification, or recommended clinical management (revascularization versus medical therapy) are resolved by subsequent consensus review between the site and CORE labs, and recorded on the corresponding CRFs

QA-CORE lab for CTA is established at the University of Ottawa Heart Institute, and the QA-CORE lab for ICA is at the Montreal Heart Institute.
